# Supplementary material for: Systemically Identifying Triple-Negative Breast Cancer Subtype-Specific Prognosis Signatures, Based on Single-Cell RNA-Seq Data
Source: Cells. 2023 Jan 19;12(3):367. doi: 10.3390/cells12030367 (PMC9913740; doi:10.3390/cells12030367)
Supplement: Supplementary file 1 [file cells-12-00367-s001.zip › Supplementary Figure S1.pdf]

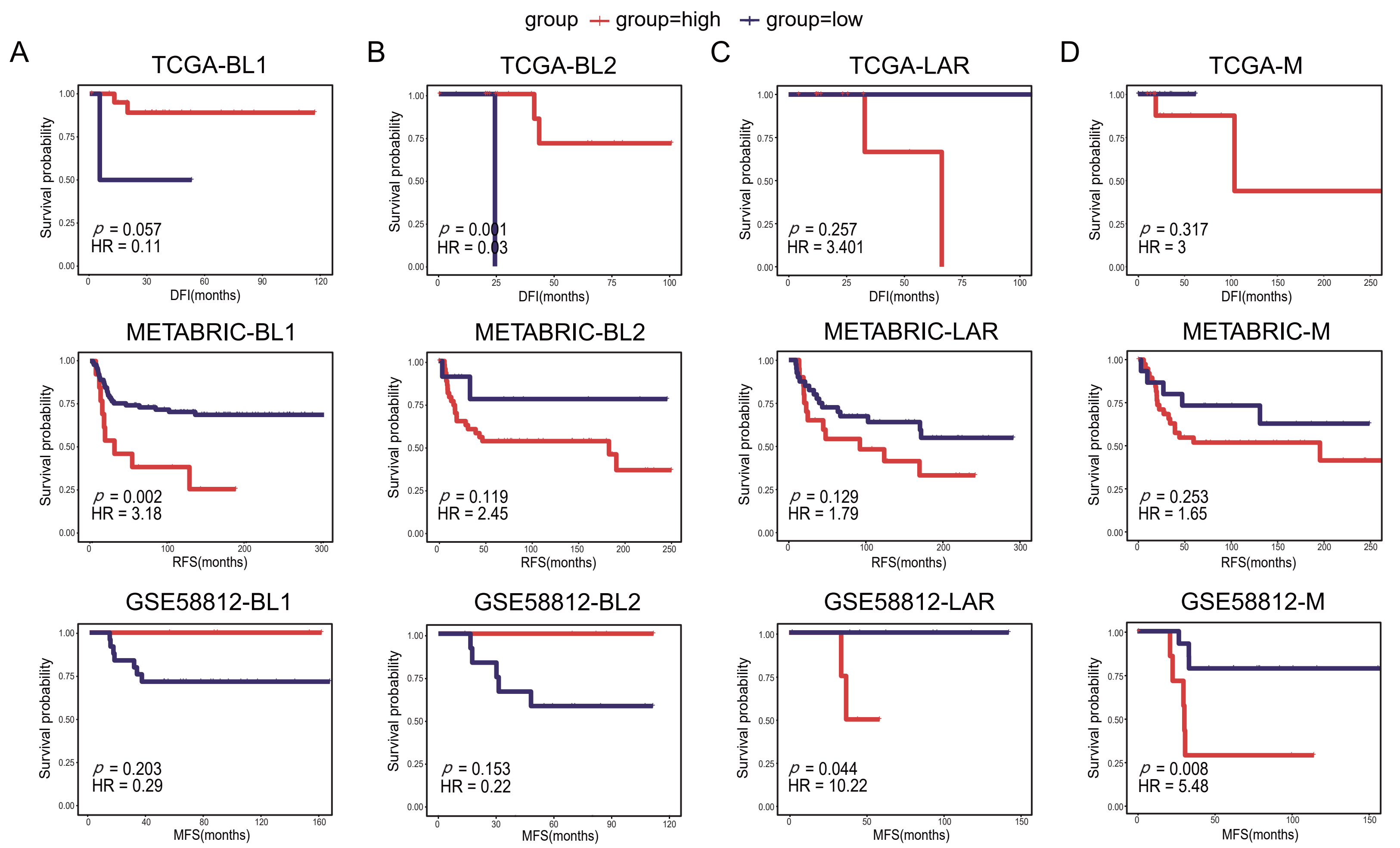

group
group=high
group=low
A
TCGA-BL1
p = 0.057
HR = 0.11
DFI(months)
B
TCGA-BL2
p = 0.001
HR = 0.03
DFI(months)
C
TCGA-LAR
p = 0.257
HR = 3.401
DFI(months)
D
TCGA-M
p = 0.317
HR = 3
DFI(months)
METABRIC-BL1
p = 0.002
HR = 3.18
RFS(months)
METABRIC-BL2
p = 0.119
HR = 2.45
RFS(months)
METABRIC-LAR
p = 0.129
HR = 1.79
RFS(months)
METABRIC-M
p = 0.253
HR = 1.65
RFS(months)
GSE58812-BL1
p = 0.203
HR = 0.29
MFS(months)
GSE58812-BL2
p = 0.153
HR = 0.22
MFS(months)
GSE58812-LAR
p = 0.044
HR = 10.22
MFS(months)
GSE58812-M
p = 0.008
HR = 5.48
MFS(months)
Survival probability
