# Supplementary material for: Systemically Identifying Triple-Negative Breast Cancer Subtype-Specific Prognosis Signatures, Based on Single-Cell RNA-Seq Data
Source: Cells. 2023 Jan 19;12(3):367. doi: 10.3390/cells12030367 (PMC9913740; doi:10.3390/cells12030367)
Supplement: Supplementary file 1 [file cells-12-00367-s001.zip › Supplementary Table S1.pdf]

Table S1. Gene composition of each TNBC subtype-specific prognosis signature (TSPSig)

BL1-TSPSig	CD99, RHOB, NFIB, UCHL1
BL2-TSPSig	EPCAM, CALML5, CD24, S100A9, S100A16, LGALS1
LAR-TSPSig	ITM2B, CYBA, TFAP2B, AR, SPDEF, MUCL1
M-TSPSig	IDH2, AZGP1, MIF, FAM3C, S100A4, C6orf15
