# Supplementary material for: Systemically Identifying Triple-Negative Breast Cancer Subtype-Specific Prognosis Signatures, Based on Single-Cell RNA-Seq Data
Source: Cells. 2023 Jan 19;12(3):367. doi: 10.3390/cells12030367 (PMC9913740; doi:10.3390/cells12030367)
Supplement: Supplementary file 1 [file cells-12-00367-s001.zip › Supplementary Table S2.pdf]

Table S2. Enrichment analysis results.

Canonical pathways	Size	*p*-Value	Count
BL1-TSPSig			
REACTOME_RHO_GTPASES_ACTIVATE_CIT	19	0.006	1
REACTOME_RHO_GTPASES_ACTIVATE_ROCKS	19	0.006	1
REACTOME_SEMA4D_INDUCED_CELL_MIGRATION_AND_GROWTH_CONE_COLLAPSE	20	0.006	1
REACTOME_RNA_POLYMERASE_III_TRANSCRIPTION_TERMINATION	23	0.007	1
REACTOME_SEMA4D_IN_SEMAPHORIN_SIGNALING	24	0.007	1
PID_ALPHA_SYNUCLEIN_PATHWAY	32	0.010	1
REACTOME_GPVI_MEDIATED_ACTIVATION_CASCADE	35	0.010	1
REACTOME_RNA_POLYMERASE_III_TRANSCRIPTION	41	0.012	1
PID_HNF3A_PATHWAY	44	0.013	1
WP_HEPATITIS_C_AND_HEPATOCELLULAR_CARCINOMA	56	0.017	1
WP_PROTEASOME_DEGRADATION	62	0.018	1
REACTOME_SEMAPHORIN_INTERACTIONS	64	0.019	1
WP_PATHWAYS_AFFECTED_IN_ADENOID_CYSTIC_CARCINOMA	65	0.019	1
PID_P75_NTR_PATHWAY	68	0.020	1
REACTOME_RHOB_GTPASE_CYCLE	70	0.021	1
WP_PARKINSONS_DISEASE_PATHWAY	71	0.021	1
REACTOME_G_ALPHA_12_13_SIGNALLING_EVENTS	80	0.024	1
WP_ANDROGEN_RECEPTOR_SIGNALING_PATHWAY	91	0.027	1
REACTOME_RHO_GTPASES_ACTIVATE_PKNS	94	0.028	1
PID_CXCR4_PATHWAY	100	0.030	1
WP_NEURAL_CREST_DIFFERENTIATION	101	0.030	1
REACTOME_UCH_PROTEINASES	102	0.030	1
WP_GASTRIN_SIGNALING_PATHWAY	115	0.034	1

KEGG_LEUKOCYTE_TRANSENDOTHELIAL_MIGRATION	116	0.034	1
WP_SPINAL_CORD_INJURY	117	0.035	1
WP_EBOLA_VIRUS_INFECTION_IN_HOST	129	0.038	1
KEGG_PARKINSONS_DISEASE	130	0.038	1
KEGG_CELL_ADHESION_MOLECULES_CAMS	133	0.039	1
REACTOME_RHO_GTPASES_ACTIVATE_FORMINS	140	0.041	1
BL2-TSPSig			
WP_BURN_WOUND_HEALING	113	0.001	2
REACTOME_IRAK4_DEFICIENCY_TLR2_4	18	0.008	1
NABA_SECRETED_FACTORS	343	0.009	2
REACTOME_REGULATION_OF_TLR_BY_ENDOGENOUS_LIGAND	21	0.009	1
PID_TOLL_ENDOGENOUS_PATHWAY	24	0.011	1
REACTOME_RHO_GTPASES_ACTIVATE_NADPH_OXIDASES	24	0.011	1
WP_IL1_AND_MEGAKARYOCYTES_IN_OBESITY	24	0.011	1
PID_RAS_PATHWAY	30	0.013	1
REACTOME_DISEASES_OF_IMMUNE_SYSTEM	31	0.014	1
REACTOME_NEUTROPHIL_DEGRANULATION	479	0.017	2
WP_RENINANGIOTENSINALDOSTERONE_SYSTEM_RAAS	44	0.020	1
PID_MYC_REPRESS_PATHWAY	63	0.028	1
KEGG_GLIOMA	65	0.029	1
WP_MELANOMA	68	0.030	1
KEGG_LONG_TERM_POTENTIATION	70	0.031	1
KEGG_PHOSPHATIDYLINOSITOL_SIGNALING_SYSTEM	76	0.034	1
REACTOME_ANTIMICROBIAL_PEPTIDES	97	0.043	1
KEGG_GNRH_SIGNALING_PATHWAY	101	0.045	1
KEGG_MELANOGENESIS	101	0.045	1

REACTOME_ANTIGEN_PROCESSING_CROSS_PRESENTATION	106	0.047	1
KEGG_OOCYTE_MEIOSIS	113	0.049	1
LAR-TSPSig			
PID_AR_TF_PATHWAY	53	0.000	2
REACTOME_SUMOYLATION	187	0.003	2
REACTOME_NEGATIVE_REGULATION_OF_ACTIVITY_OF_TFAP2_AP_2_FAMILY_TRANSCRIPTION_FACTORS	10	0.004	1
REACTOME_ACTIVATION_OF_THE_TFAP2_AP_2_FAMILY_OF_TRANSCRIPTION_FACTORS	12	0.005	1
REACTOME_KILLING_MECHANISMS	12	0.005	1
REACTOME_TFAP2_AP_2_FAMILY_REGULATES_TRANSCRIPTION_OF_GROWTH_FACTORS_AND_THEIR_RECEPTORS	15	0.007	1
REACTOME_DEFECTIVE_GALNT3_CAUSES_HFTC	16	0.007	1
REACTOME_DEFECTIVE_C1GALT1C1_CAUSES_TNPS	17	0.008	1
REACTOME_RHO_GTPASE_EFFECTORS	324	0.008	2
REACTOME_SUMOYLATION_OF_TRANSCRIPTION_FACTORS	20	0.009	1
REACTOME_TERMINATION_OF_O_GLYCAN_BIOSYNTHESIS	23	0.010	1
REACTOME_RHO_GTPASES_ACTIVATE_NADPH_OXIDASES	24	0.011	1
REACTOME_RUNX2_REGULATES_OSTEOBLAST_DIFFERENTIATION	24	0.011	1
REACTOME_DECTIN_2_FAMILY	26	0.012	1
REACTOME_SUMOYLATION_OF_INTRACELLULAR_RECEPTORS	30	0.013	1
PID_AR_NONGENOMIC_PATHWAY	31	0.014	1
REACTOME_RUNX2_REGULATES_BONE_DEVELOPMENT	31	0.014	1
REACTOME_ROS_AND_RNS_PRODUCTION_IN_PHAGOCYTES	36	0.016	1
REACTOME_DETOXIFICATION_OF_REACTIVE_OXYGEN_SPECIES	37	0.017	1
WP_GENETIC_CAUSES_OF_PSVDINCPH	37	0.017	1
REACTOME_TRANSCRIPTIONAL_REGULATION_BY_THE_AP_2_TFAP2_FAMILY_OF_TRANSCRIPTION_FA	38	0.017	1

CTORS			
WP_NUCLEAR_RECEPTORS	38	0.017	1
WP_MICROGLIA_PATHOGEN_PHAGOCYTOSIS_PATHWAY	40	0.018	1
PID_HNF3A_PATHWAY	44	0.020	1
PID_HES_HEY_PATHWAY	48	0.021	1
WP_MIRNA_REGULATION_OF_PROSTATE_CANCER_SIGNALING_PATHWAYS	49	0.022	1
REACTOME_NUCLEAR_RECEPTOR_TRANSCRIPTION_PATHWAY	53	0.024	1
PID_RAC1_PATHWAY	54	0.024	1
REACTOME_HSP90_CHAPERONE_CYCLE_FOR_STEROID_HORMONE_RECEPTORS_SHR_IN_THE_PRESENCE_OF_LIGAND	57	0.025	1
WP_PREIMPLANTATION_EMBRYO	59	0.026	1
PID_AR_PATHWAY	61	0.027	1
REACTOME_O_LINKED_GLYCOSYLATION_OF_MUCINS	62	0.028	1
REACTOME_ACTIVATED_PKN1_STIMULATES_TRANSCRIPTION_OF_AR_ANDROGEN_RECEPTOR_REGULATED_GENES_KLK2_AND_KLK3	67	0.030	1
REACTOME_DISEASES_ASSOCIATED_WITH_O_GLYCOSYLATION_OF_PROTEINS	68	0.030	1
KEGG_LEISHMANIA_INFECTION	72	0.032	1
PID_BETA_CATENIN_NUC_PATHWAY	80	0.035	1
PID_SMAD2_3NUCLEAR_PATHWAY	82	0.036	1
REACTOME_RAC2_GTPASE_CYCLE	88	0.039	1
KEGG_PROSTATE_CANCER	89	0.039	1
WP_ANDROGEN_RECEPTOR_SIGNALING_PATHWAY	91	0.040	1
WP_TNFALPHA_SIGNALING_PATHWAY	92	0.041	1
REACTOME_RAC3_GTPASE_CYCLE	94	0.041	1
REACTOME_RHO_GTPASES_ACTIVATE_PKNS	94	0.041	1
WP_NEURAL_CREST_DIFFERENTIATION	101	0.045	1

REACTOME_ANTIGEN_PROCESSING_CROSS_PRESENTATION	106	0.047	1
REACTOME_SIGNALING_BY_VEGF	106	0.047	1
REACTOME_AMYLOID_FIBER_FORMATION	110	0.048	1
REACTOME_O_LINKED_GLYCOSYLATION	111	0.049	1
KEGG_OOCYTE_MEIOSIS	113	0.049	1
M-TSPSig			
KEGG_PHENYLALANINE_METABOLISM	18	0.007	1
WP_TCA_CYCLE_AKA_KREBS_OR_CITRIC_ACID_CYCLE	18	0.007	1
REACTOME_CITRIC_ACID_CYCLE_TCA_CYCLE	22	0.008	1
REACTOME_MISCELLANEOUS_TRANSPORT_AND_BINDING_EVENTS	26	0.010	1
KEGG_CITRATE_CYCLE_TCA_CYCLE	31	0.012	1
REACTOME_GENE_AND_PROTEIN_EXPRESSION_BY_JAK_STAT_SIGNALING_AFTER_INTERLEUKIN_12_STIMULATION	38	0.014	1
KEGG_TYROSINE_METABOLISM	42	0.016	1
WP_METABOLIC_REPROGRAMMING_IN_COLON_CANCER	42	0.016	1
REACTOME_INTERLEUKIN_12_SIGNALING	47	0.017	1
KEGG_GLUTATHIONE_METABOLISM	50	0.019	1
REACTOME_PYRUVATE_METABOLISM_AND_CITRIC_ACID_TCA_CYCLE	55	0.020	1
REACTOME_TRANSCRIPTIONAL_ACTIVATION_OF_MITOCHONDRIAL_BIOGENESIS	55	0.020	1
REACTOME_INTERLEUKIN_12_FAMILY_SIGNALING	57	0.021	1
KEGG_PEROXISOME	78	0.029	1
WP_HAIR_FOLLICLE_DEVELOPMENT_CYTODIFFERENTIATION_PART_3_OF_3	87	0.032	1
REACTOME_MITOCHONDRIAL_BIOGENESIS	94	0.035	1
WP_SPINAL_CORD_INJURY	117	0.043	1
WP_ADIPOGENESIS	131	0.048	1
REACTOME_RESPONSE_TO_ELEVATED_PLATELET_CYTOSOLIC_CA2	132	0.048	1

TSPSig, TNBC subtype-specific prognosis signature.
