# Supplementary material for: Systemically Identifying Triple-Negative Breast Cancer Subtype-Specific Prognosis Signatures, Based on Single-Cell RNA-Seq Data
Source: Cells. 2023 Jan 19;12(3):367. doi: 10.3390/cells12030367 (PMC9913740; doi:10.3390/cells12030367)
Supplement: Supplementary file 1 [file cells-12-00367-s001.zip › Supplementary Table S3.pdf]

Table S3. Univariate and multivariate Cox regression analyses of RFS in each TNBC subtype

	Univariate analysis			Multivariate analysis		
Variables	HR	95% CI	*p*-Value	HR	95% CI	*p*-Value
BL1-subtype						
BL1-TSPSig (high vs low)	3.077	1.341-7.062	0.008 **	4.136	1.678-10.198	0.002 **
Chemotherapy (yes vs no)	1.478	0.664-3.294	0.339	1.482	0.500-4.396	0.478
Age	0.991	0.961-1.022	0.573	0.982	0.943-1.022	0.368
Tumor_size	1.024	0.997-1.052	0.085.	1.030	0.994-1.068	0.106
Stage (III vs I/II)	1.488	0.352-6.290	0.589	1.067	0.179-6.365	0.944
BL2-subtype						
BL2-TSPSig (high vs low)	4.815	0.632-36.684	0.129	3.851	0.457-32.457	0.215
Chemotherapy (yes vs no)	1.130	0.397-3.213	0.819	0.419	0.110-1.603	0.204
Age	1.004	0.963-1.046	0.860	0.989	0.941-1.040	0.674
Tumor_size	1.031	1.009-1.055	0.007 **	1.025	0.999-1.052	0.062.
Stage (III vs I/II)	5.313	1.840-15.350	0.002 **	3.585	1.082-11.878	0.037 *
LAR-subtype						
LAR-TSPSig (high vs low)	3.170	1.365-7.363	0.007 **	4.092	1.641-10.202	0.003 **
Chemotherapy (yes vs no)	2.092	0.903-4.850	0.085.	1.408	0.518-3.827	0.502
Age	0.987	0.954-1.022	0.472	0.968	0.924-1.014	0.168
Tumor_size	1.023	1.006-1.041	0.008 **	1.018	1.000-1.036	0.047 *
Stage (III vs I/II)	3.020	1.103-8.266	0.032 *	2.067	0.625-6.835	0.234
M-subtype						
M-TSPSig (high vs low)	2.232	0.645-7.722	0.205	2.222	1.456-8.326	0.236
Chemotherapy (yes vs no)	1.752	0.693-4.435	0.236	0.888	0.231-3.416	0.863
Age	0.956	0.917-0.997	0.034 *	0.950	0.894-1.009	0.095.
Tumor_size	1.020	0.994-1.048	0.135	1.017	0.989-1.045	0.240
Stage (III vs I/II)	10.380	2.079-51.830	0.004 **	5.650	0.976-32.706	0.053.

TSPSig, the TNBC subtype-specific prognosis signature; **, $p < 0.01$; *, $0.01 \leq p < 0.05$; ., $0.05 \leq p < 0.1$.
