# Supplementary material for: Systemically Identifying Triple-Negative Breast Cancer Subtype-Specific Prognosis Signatures, Based on Single-Cell RNA-Seq Data
Source: Cells. 2023 Jan 19;12(3):367. doi: 10.3390/cells12030367 (PMC9913740; doi:10.3390/cells12030367)
Supplement: Supplementary file 1 [file cells-12-00367-s001.zip › Supplementary Table S4.pdf]

Table S4. Summary of TNBC subtype patients with chemotherapy in three validation datasets.

	Total	BL1	BL2	LAR	M	Others
TCGA	83	22	14	9	18	20
METABRIC	157	67	29	26	21	14
GSE96058	104	38	13	8	23	22
