# Supplementary material for: Systemically Identifying Triple-Negative Breast Cancer Subtype-Specific Prognosis Signatures, Based on Single-Cell RNA-Seq Data
Source: Cells. 2023 Jan 19;12(3):367. doi: 10.3390/cells12030367 (PMC9913740; doi:10.3390/cells12030367)
Supplement: Supplementary file 1 [file cells-12-00367-s001.zip › Supplementary Table S5.pdf]

Table S5. Summary information of TNBC subtype cell lines.

**Subtypes**	**Cell lines**
BL1	HCC1187, HCC1143, HCC1937, COLO-824, HCC1599, HCC2157
BL2	CAL-85-1, HCC1806, HCC70, HDQ-P1, MDA-MB-231, MDA-MB-436, MDA-MB-468, MDA-MB-157
LAR	CAL-148, MDA-MB-453, MFM-223, MRK-nu-1
M	CAL-120, CAL-51, HCC1395, HCC38, Hs-578-T, BT-549
Others	DU-4475, BT-20
