# Supplementary figures and images for: Systemically Identifying Triple-Negative Breast Cancer Subtype-Specific Prognosis Signatures, Based on Single-Cell RNA-Seq Data
Source: Cells. 2023 Jan 19;12(3):367. doi: 10.3390/cells12030367 (PMC9913740; doi:10.3390/cells12030367)

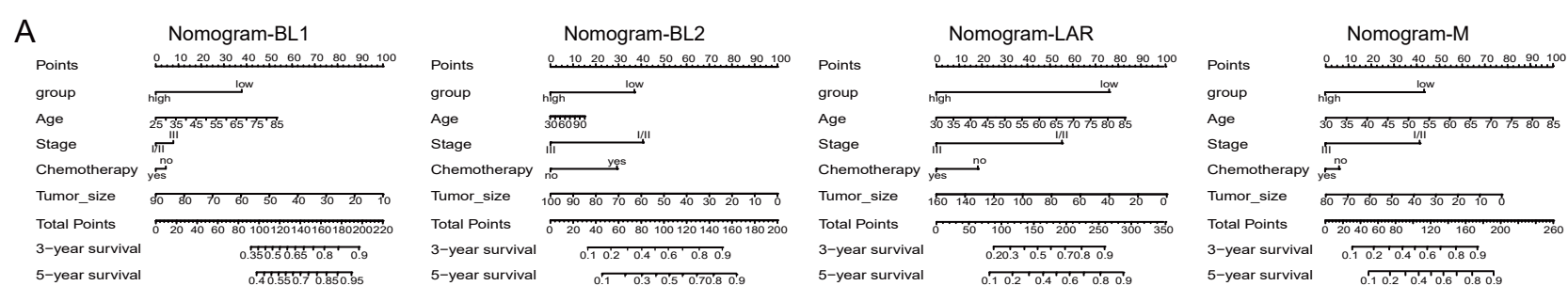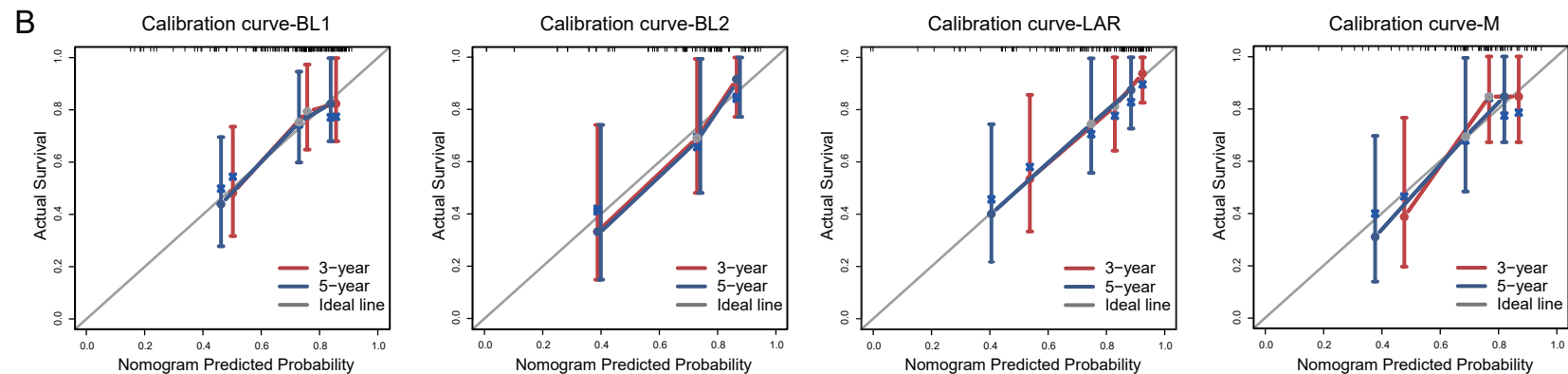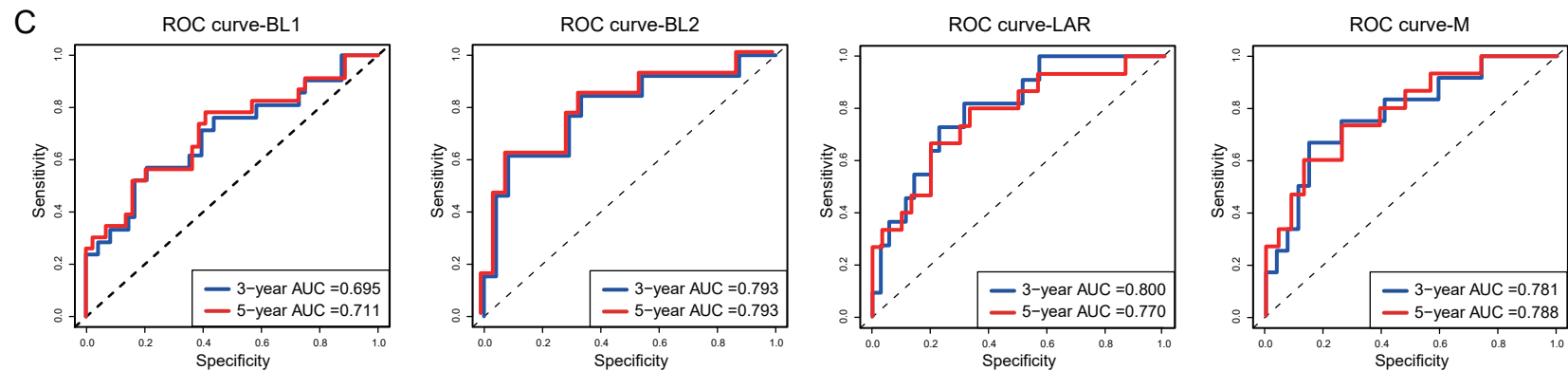

Supplement: Supplementary file 1 [file cells-12-00367-s001.zip › Supplementary Figure S2.pdf]
